# A new species of the endemic Himalayan genus *Liurana* (Anura, Ceratobatrachidae) from southeastern Tibet, China, with comments on the distribution, reproductive biology, and conservation of the genus

**DOI:** 10.24272/j.issn.2095-8137.2019.025

**Published:** 2019-05-18

**Authors:** Ke Jiang, Kai Wang, Yu-Fan Wang, Cheng Li, Jing Che

**Affiliations:** 1 State Key Laboratory of Genetic Resources and Evolution, Kunming Institute of Zoology, Chinese Academy of Sciences, Kunming Yunnan 650223, China; 2 Sam Noble Oklahoma Museum of Natural History and Department of Biology, University of Oklahoma, Norman OK 73072-7029, USA; 3 Zhejiang Forest Resource Monitoring Center, Hangzhou Zhejiang 310020, China; 4 Funsome Nature Center, Shenzhen Guangdong 518067, China; 5 Southeast Asia Biodiversity Research Institute, Chinese Academy of Sciences, Yezin Nay Pyi Taw 05282, Myanmar

**Keywords:** Advertisement call, Biogeography, Ecology, Natural history, Tibet, Taxonomy

## Abstract

A new species of the genus *Liurana* Dubois, 1986 is described from Medog County, Tibet, China, based on morphological and molecular data. The new species can be differentiated from all other congeners by the following combination of characters: (1) head wider than long; (2) tympanum distinct and large; (3) hindlimb long, tibiotarsal articulation beyond tip of snout when adpressed; (4) belly with flat tubercles, cloacal region with small tubercles; (5) transverse bands distinctly on dorsal limbs, four bands on thigh and three on tibia; and, (6) dark brown marbled patterns or speckles on white belly. Here, we also discuss the distribution pattern of *Liurana* in the East Himalaya region, the role of the Yarlung Tsangpo River in the speciation and genetic isolation of congeners, the direct developmental mode of reproduction, and the two different ecotypes of the genus. Lastly, we provide conservation recommendations for the genus in southeastern Tibet.

## INTRODUCTION

As a wide-spread amphibian family in Southeast Asia, species of the family Ceratobatrachidae are distributed from the southern foothills of the Himalaya to the tropics of Southeast Asia (Yan et al., 2016). Members of the family are characterized as tropical specialists, with many species undergoing direct development without reliance on standing water bodies for breeding (Brown & Alcala, 1982; Brown et al., 2015). Within this family, frogs of the genus *Liurana* represent an understudied yet fascinating endemic group from the East Himalaya region.

First considered as a subgenus of *Ingerana* by Dubois (1986), *Liurana* was established based on the type species *Cornufer xizangensis* Hu, 1987, with the subgenus later elevated to full genus based on morphological evidence (Fei et al., 1997). This taxonomic elevation is supported by recent phylogenetic studies, where *Liurana* was recovered as a distinct monophyletic clade from *Ingerana*, *Platymantis*, and *Cornufer* (Yan et al., 2016). However, despite research efforts on the higher-level systematic relationships of the genus *Liurana*, little attention has been given to the species level taxonomy of the group in the Himalaya. To date, only a few studies have focused on species level taxonomy of the genus, with just a single study conducted in the last decade (Borah et al., 2013; Fei et al., 1997; Huang & Ye, 1997; Sichuan Biological Research Institute, 1977). As a result, all recognized species of *Liurana* are known from only a few vouchered specimens, and little is understood about their morphological variation and population structure. Based on limited studies, three species have been recognized in the genus to date, including *L. alpina* Huang, Ye, 1997, *L. medogensis* Fei, Ye, Huang, 1997, and *L. xizangensis* (Hu, 1987).

During herpetological surveys in southeastern Tibet from 2012 to 2016, 20 specimens of the genus *Liurana* were collected from Bomê and Medog Counties of the Nyingchi Prefecture, Tibet, China. Combining phylogenetic and morphological datasets, we describe here a new species of the genus from the tropical rainforest of Medog County, Nyingchi Prefecture, Tibet, China. Furthermore, we comment on the evolution, ecology, natural history, and conservation of the genus *Liurana* in China.

## MATERIALS AND METHODS

### Taxon sampling

**Figure 1:**
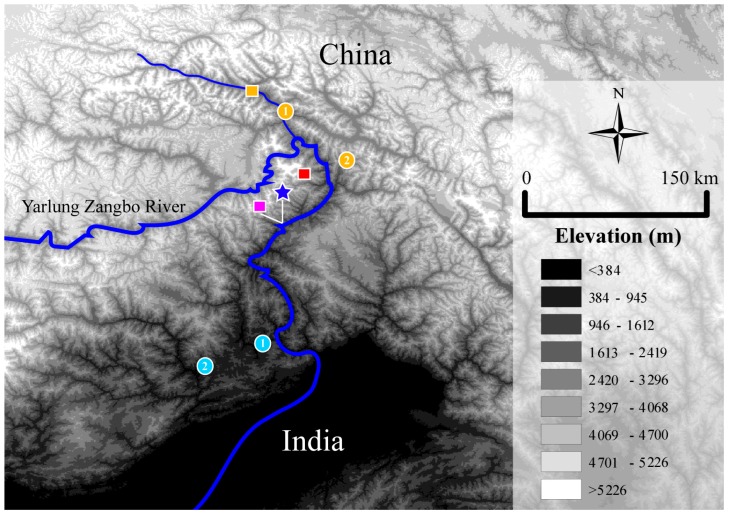
Distribution of *Liurana* species in the East Himalaya

A total of 20 individuals of the recognized species of the genus *Liurana* were collected from different localities in the Bomê and Medog Counties, Nyingchi Prefecture, southeastern Tibet, China, and were comprised of 18 individuals of *L. alpina*, *L. medogensis*, and *L. xizangensis* and two individuals (one adult male and one adult female) of the new species (Figure 1; Appendix I). Following euthanasia, tissue samples were taken and preserved in 95% ethanol, with the specimens then fixed in 10% buffered formalin solution and transferred to 75% ethanol after fieldwork. All specimens were deposited in the Museum of the Kunming Institute of Zoology, Chinese Academy of Sciences (KIZ) (Appendix II).

Blue star represents type locality of the new species, *Liurana vallecula* **sp. nov**. Rectangles represent type localities of recognized species and circles represent additional known localities of recognized species. Different species are color-coded as follows: *L. alpina* (red), *L. medogensis* (purple), and *L. xizangensis* (yellow). For *L. xizangensis*, the two additional localities are (1) Tongmai, Bomê County, Tibet, China; and (2) 62K, Medog County, Tibet, China. The two light blue localities represent reported records of *Liurana* cf. *medogensis* from Southern Tibet (see discussion for details).

### Morphological data

We confirmed the sex of each specimen by anatomical observation, with an incision made on the left side. All measurements were carried out by Ke Jiang using a digital caliper to the nearest 0.1 mm. Morphological characters and their measurement followed Fei et al. (2009) and included: snout–vent length (SVL); head length (HL), measured from posterior corner of mandible to tip of snout; head width (HW), measured at the angle of the jaw; snout length (SL), measured from tip of snout to anterior corner of eye; internarial distance (IND); interorbital distance (IOD), measured at the shortest distance between upper eyelid; maximum width of upper eyelid (UEW); eye diameter (ED), measured as the horizontal diameter of eye); tympanum diameter (TD), measured as the maximum horizontal diameter of tympanum); length of lower arm and hand (LAHL), measured from the elbow joint to the tip of the longest finger; largest diameter of lower arm (LAD); hand length (HAL), measured from the base of the outer metacarpal tubercle to the tip of finger III; femur length (FML), measured as the linear distance between the insertion of the leg to the knee joint; tibia length (TL), measured as the linear distance between the knee joint and tibiotarsal articulation; length of tarsus and foot (TFL), measured from the tibiotarsal articulation to the tip of toe IV; and foot length (FL), measured from the base of the inner metatarsal tubercle to the tip of toe IV.

In addition to the newly obtained data, morphological data of congeners were also obtained from published literature for comparison (Borah et al., 2013; Fei et al., 1997, 2009; Huang & Ye, 1997).

### Molecular analysis

Genomic DNA was extracted from tissue samples using standard phenol-chloroform protocols (Sambrook et al., 1989). Fragments of a single mitochondrial DNA locus (cytochrome c oxidase subunit I, *COI*) and three nuclear loci, including recombination activating protein 1 (Rag1), tyrosinase (Tyr), and rhodopsin (Rhod), were targeted and amplified following published primers and protocols (Che et al., 2011; Yan et al., 2016). The products were purified with a Gel Extraction Mini Kit (Watson Biotechnologies, Shanghai, China) and sequenced on an ABI 3730xI DNA automated sequencer (Applied Biosystems, UK).

Additional sequences of congeners and closely related outgroups were obtained through GenBank (Appendix II). Sequences were aligned using MUSCLE implemented in Geneious R10.0.6. Uncorrected genetic distances of the mitochondrial locus *COI* were calculated using PAUP* v4.0b10. To assess phylogenetic congruence between the mitochondrial and nuclear data, the phylogeny for each gene was inferred independently using Bayesian analyses. As no strongly supported incongruences between mitochondrial and nuclear data were observed visually, two datasets were concatenated for the final analyses.

Phylogenetic relationships were inferred using both partitioned Bayesian (MrBayes v3.2.1; Ronquist & Huelsenbeck, 2003) and maximum likelihood analyses (RAXML VI-HPC v8.2.10; Stamatakis, 2014). For Bayesian analyses, all protein-coding genes were partitioned by codon positions, and the best models of nucleotide substitution were selected for each partition by the Akaike Information Criterion (AIC), as implemented in jModelTest v2.1.10 (Darriba et al., 2012; Guindon & Gascuel, 2003). A rate multiplier model was used to allow substitution rates to vary among subsets. Default settings were used for all other model parameters. Two independent Markov chain Monte Carlo analyses were run, each with four Metropolis-coupled chains, a melting temperature of 0.02, and an exponential distribution with a rate parameter of 25 as the prior branch lengths (Marshall, 2010). All Bayesian analyses were run for 6 000 000 generations, with parameters and topologies sampled every 3 000 generations. Stationarity and convergence were assessed with TRACER v1.6.0 (Rambaut et al., 2013).

Partitioned maximum likelihood analyses were conducted on the concatenated dataset using the same partitioning strategy as for Bayesian analysis. The more complex model (GTR+Γ) was applied for all subsets (Table 1), with 1 000 replicate ML inferences. Each inference was initiated with a random starting tree, and nodal support was assessed with 1 000 bootstrap pseudoreplicates (Stamatakis et al., 2008).

**Table 1:** Partition strategies and best evolutionary models selected for each partition

Gene	Codon	Fragment length (bp)	Model selected
*COI*	1st	190	GTR
	2nd	190	HKY+Γ
	3rd	190	HKY
*Rag1*	1st	184	F81
	2nd	184	HKY
	3rd	184	HKY
*Rod*	1st	105	JC
	2nd	105	F81
	3rd	105	GTR
*Tyr*	1st	177	GTR+Γ
	2nd	177	HKY+Γ
	3rd	177	HKY+Γ

The resulting phylogenetic trees were rooted using the clade containing *Ingerana* and *Limnonectes*, following recent studies on phylogenetic relationships of the focal group (Yan et al., 2016).

## RESULTS

### Morphology

Morphometric variation of the examined *Liurana* species is summarized in Table 2. The unidentified specimens of *Liurana* were morphologically most similar to *L. medogensis* and could be differentiated from all recognized species by having a relatively wider head (HW/HL >100% vs. <100%) and longer hindlimbs, with tibiotarsal articulation reaching beyond tip of snout when adpressed (vs. reaching anterior corner of eye only). Furthermore, the unidentified *Liurana* specimens from Medog possessed distinct tubercles near the cloaca, which are absent or indistinct in all recognized congeners except for *L. xizangensis*.

**Table 2:** Morphological comparisons for four species of the genus *Liurana*

	*Liurana vallecula* sp. nov.	*L. alpina*	*L. medogensis*	*L. xizangensis*
SVL (mm)	14.6 (1) 20.4 (1)	23.2–24.9 (3) 25.5 (1)	13.1–19.0 (3)	20.6–22.5 (9) 29.4–30.5 (2)
HL/HW	<100%	>100%	>100%	>100%
Tibiotarsal articulation	Beyond tip of snout	Reaching anterior corner of eye	Reaching anterior corner of eye	Reaching anterior corner of eye
Flat tubercles on belly	Absent	Absent	Absent	Present
Tubercles around cloaca	Present	Absent	Indistinct	Present
Ventral pattern	Thin, marbled-patterns	Thin, marbled-patterns	Thick, vermiculated stripes	Thin, marbled-patterns

Morphological abbreviations are listed in the methods.

Coloration and ornamentation were highly variable among the examined specimens of *L. alpina* and *L. xizangensis*, ranging from uniform bright orange reddish to brownish gray with blackish speckles (Figure 3). For *L. medogensis* and the two unidentified individuals from Medog, the coloration and ornamentation were less variable. A single individual of *L. medogensis* (KIZ05587) possessed a broad, yellowish dorsal vertebral stripe from snout to vent, whereas the other individuals of the same species exhibited a much darker vertebral stripe in light reddish brown.

### Phylogeny

**Figure 2:**
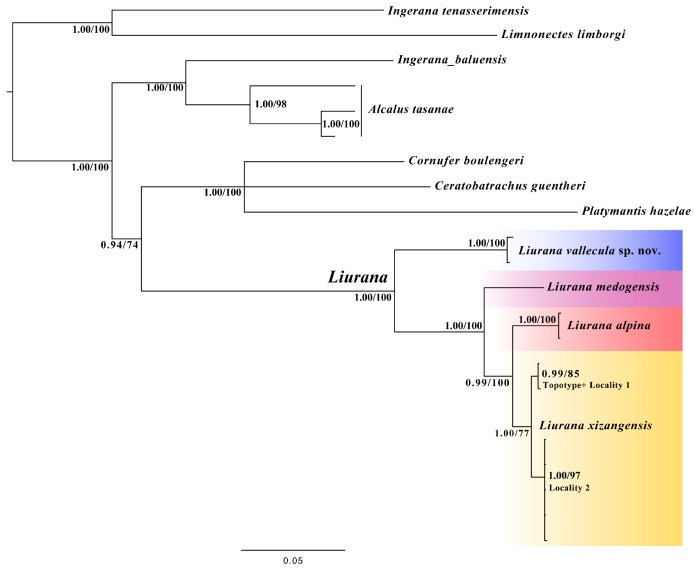
Phylogenetic relationships among *Liurana* species based on maximum likelihood and Bayesian analyses of one mitochondrial gene (*COI*) and three nuclear genes (*Rag1*, *Rod*, and *Tyr*)

The phylogenetic placement of the genus *Liurana* is similar to previous results (Yan et al., 2016), with the genus recovered as monophyletic with strong support (Bayesian posterior probability (PP)=1.00; ML bootstrap support (BS)=100) (Figure 2). Within the genus, *L. xizangensis* and *L. alpina* form a monophyletic group with strong supports (PP=0.99, BS=100), which is sister to *L. medogensis* (PP=1.00, BS=100). The two unidentified individuals (*Liurana* sp.) collected from Medog County form a distinct, monophyletic clade (PP=1.00, BS=100), which is basal with respect to all other *Liurana* congeners (Figure 2).

Maximum likelihood bootstrap and Bayesian posterior probability values are given at all nodes (in such order respectively), except the internal nodes within *L. xizangensis* from Locality #2, which have short branch lengths and resemble polytomy.

For the obtained 570 bp fragment of *COI* data, the uncorrected genetic distance between the two unidentified *Liurana* individuals is 0.88% only, while such distance is 22.19%–22.22% to its morphologically most similar congener, *L. medogensis*, 21.67%–22.63% to *L. alpina*, and 20.19%–21.30% to *L. xizangensis* (Table 3). The observed intraspecific variation among recognized *Liurana* species is 0%–0.183% within conspecifics of *L. alpina*. For *L. xizangensis*, the greatest genetic divergence (3.12%) is observed between individuals from Tongmai, Bomê County, Tibet, China (Locality #1) and 62K, Medog County, Tibet, China (Locality #2), which are on different sides of the Yarlung Tsangpo River (Figure 1).

**Table 3:** Uncorrected genetic distances of the *COI* fragment obtained (570 bp) among *Liurana* species

1	*L.* sp. nov. YPX47504	–													
2	*L.* sp. nov. YPX47527	0.00877	–												
3	*L. medogensis* KIZ 010955	0.22191	0.222	–											
4	*L. alpina* KIZ 07357	0.22029	0.21666	0.13394	–										
5	*L. alpina* KIZ 011140	0.22632	0.22281	0.13207	0.00178	–									
6	*L. alpina* KIZ 011141	0.22216	0.21853	0.13211	0.00183	0	–								
7	*L. xizangensis* KIZ 06707 (Locality #2)	0.2129	0.21297	0.14312	0.09541	0.09345	0.09358	–							
8	*L. xizangensis* KIZ 09956 (Locality #2)	0.2129	0.21297	0.14312	0.09541	0.09345	0.09358	0	–						
9	*L. xizangensis* KIZ 012705 (Locality #2)	0.2129	0.21297	0.14312	0.09541	0.09345	0.09358	0	0	–					
10	*L. xizangensis* KIZ 012706 (Locality #2)	0.2129	0.21297	0.14312	0.09541	0.09345	0.09358	0	0	0	–				
11	*L. xizangensis* KIZ 011104 (Locality #2)	0.2129	0.21297	0.14312	0.09541	0.09345	0.09358	0	0	0	0	–			
12	*L. xizangensis* KIZ 012707 (Locality #2)	0.21107	0.21114	0.14495	0.09725	0.09535	0.09541	0.00183	0.00183	0.00183	0.00183	0.00183	–		
13	*L. xizangensis* KIZ 012704 (Topotype)	0.20372	0.20379	0.12661	0.08624	0.08436	0.0844	0.02752	0.02752	0.02752	0.02752	0.02752	0.02936	–	
14	*L. xizangensis* KIZ 014046 (Locality #1)	0.20187	0.20195	0.12844	0.08807	0.0862	0.08624	0.02936	0.02936	0.02936	0.02936	0.02936	0.03119	0.00183	–

Because the two unidentified specimens of *Liurana* from Medog possess a suite of distinct morphological characteristics, high genetic divergence from congeners, and monophyletic and distinct phylogenetic position, we here describe them as a new species.

### Taxonomy

**Figure 3:**
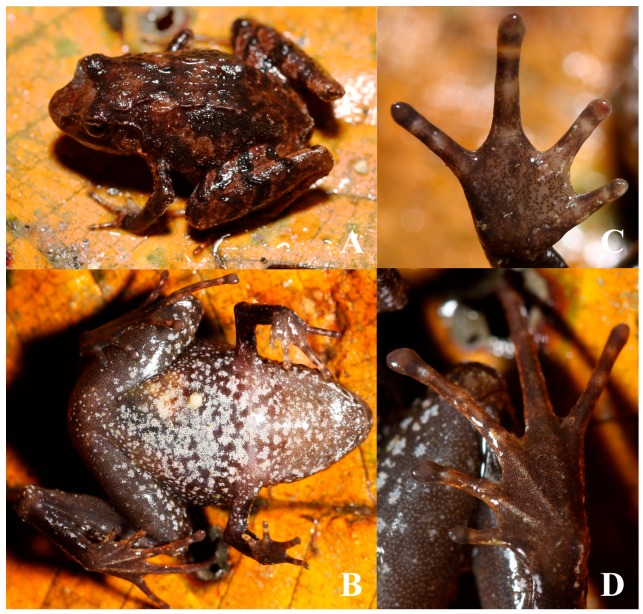
Holotype of *Liurana vallecula* sp. nov. in life (adult female, KIZ014083)

**Figure 4:**
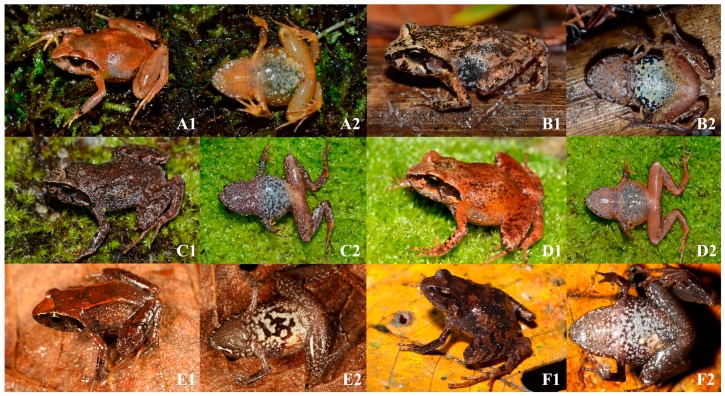
Comparisons of dorsal (1) and ventral (2) views of live individuals of *Liurana xizangensis* (A and B), *L. alpina* (C and D), *L. medogensis* (E), and *Liurana vallecula* sp. nov. (F). Photos by Ke Jiang, Kai Wang, and Yu-Fan Wan

**Figure 5:**
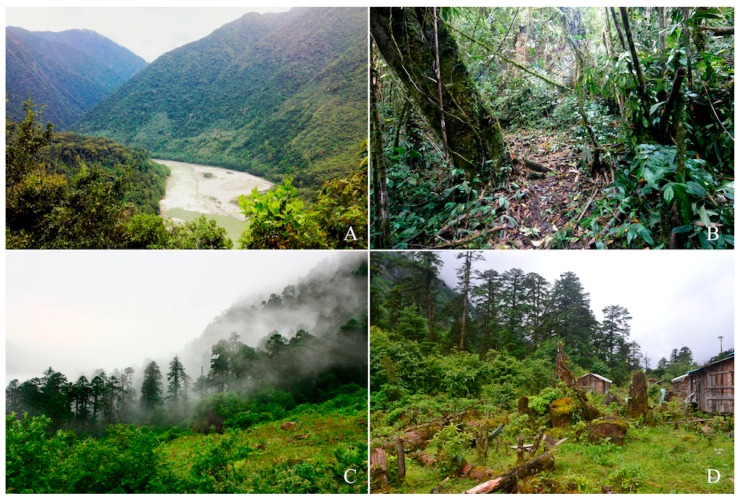
Habitat of *Liurana vallecula* sp. nov. (A) (Xirang, elevation 550 m a.s.l.), *L. medogensis* (B) (Xigong Lake, elevation 1 300 m a.s.l.), *L. alpina* (C) (Dayandong, elevation 3 000 m a.s.l.), and *L. xizangensis* (D) (62K, elevation 2 800 m a.s.l.) in Medog County, southeastern Tibet, China, respectively. Photos by Ke Jiang, Kai Wang, and Shuai Wang

*Liurana vallecula* **sp. nov.** Jiang, Wang, Wang, Li, and Che Figure 1, Figure 2, Figure 3, Figure 4 and Figure 5.

A: Dorsolateral view; B: Ventral view; C: Ventral close-up of hand; D: Ventral close-up of feet. Photos by Yu-Fan Wang.

**Holotype:** KIZ014083, adult female, from the side of Yarlung Zangbo River near Xirang (N29.1776°, E95.0057°, elevation 550 m a.s.l.; Figure 3), Beibeng, Medog County, southeastern Tibet, China. Collected by Cheng Li on 17 April 2016.

**Paratype:** KIZ014106, adult male, from the side of Baimaxilu River (a tributary of the Yarlung Zangbo River) near Maniweng (N29.2667°, E95.1667°, elevation 850 m a.s.l.), Beibeng, Medog County, southeastern Tibet, China. Collected by Ke Jiang and Yu-Fan Wang on 29 April 2016.

**Diagnosis:** *Liurana vallecula* **sp. nov.** is assigned to the genus *Liurana* by its phylogenetic position and the following morphological characters: (1) body size small (SVL 14.6–20.4 mm, *n*=2); (2) tips of fingers and toes not expanded, (3) grooves absent on tips of fingers and toes; (4) webbing absent on all digits; (5) metacarpal tubercles and metatarsal tubercles absent; (6) tarsal fold absent; and (7) vocal sac and vocal sac openings absent.

*Liurana vallecula* **sp. nov.** can be distinguished from all currently recognized congeners by a combination of the following morphological characters: (1) head wider than long HW/HL 108%–113%; (2) tympanum distinct, large; (3) hind limbs long, with tibiotarsal articulation reaching beyond tip of snout when adpressed; (4) flat tubercles present on ventral surface of body; (5) small tubercles present on cloacal region; and (6) dark brown marbled pattern present on ventral surface of body. 

**Description of holotype:** Body size small, SVL 20.4 mm. Head wider than long (HW/HL=108%); snout rounded, slightly projecting over lower jaw; canthus rostralis distinct, relatively sharp; nostril closer to tip of snout than to eye; loreal region slightly concave and oblique; eye relatively large (ED/HL=40%); internarial distance larger than interorbital distance and width of upper eyelid; tympanum oval, concealed under skin, with distinct, elevated rim, slightly less than half of eye diameter (TD/ED=45%); supratympanic fold distinct, extending posterior-inferiorly to area above forelimb insertion; pair of distinct skin folds present on head from posterior orbit dorsally to pectoral region, symmetrical along vertebral line, extending toward medial line posteriorly.

Forelimb slender; forearm and hand length less than half of body length (LAHD/SVL=44%); fingers compressed vertically, tips rounded but not expanded, transverse grooves absent, relative finger lengths I<II<IV<III; subarticular tubercles absent; three metacarpal tubercles present, flat and indistinct. Hind limb slender, tibiotarsal articulation reaching beyond tip of snout when adpressed; heels overlapped when flexed and held perpendicular to body; tibia length larger than half of body length (TL/SVL=58%); toes compressed vertically, tips slightly expanded, transverse grooves absent; relative toe lengths I<II<V<III<IV; toe webbing absent; subarticular tubercles indistinct; inner metatarsal tubercle oval, indistinct; outer metatarsal tubercle indistinct; tarsal fold absent.

Relatively weak, discontinuous folds present on dorsolateral body from shoulder to about two thirds of trunk on each side of body; single skin fold present along vertebrate from snout to vent, much weaker than discontinuous folds laterally; dorsal surface relatively rough, tubercles randomly scattered on dorsal and lateral head, body, and limbs as well as around cloaca; tubercles much finer on ventrolateral region of body. Ventral head, body, and limbs mostly smooth, except several flat tubercles on base of ventral thigh and small tubercles on ventral surface of tarsus and metatarsus. Vomerine teeth absent; tongue large, elongated oval, deeply notched posteriorly, small papillae scattered on tongue.

**Coloration of holotype in life:** The dorsal surfaces of the head and body are reddish brown. Dark brown streaks and marble patterns are present on dorsal head, body, and limbs, including a transverse streak between orbit on dorsal surface of head, a X-shaped pattern on pectoral region of dorsum, irregular marble patterns on lateral body, and transverse streaks across dorsal limbs (more distinct on the dorsal hind limbs). The lower parts of canthus rostralis and supratympanic fold are blackish brown. The ventrolateral surface of the body is dark brown, with small white spots scattered across. Ventral surfaces of limbs, head, and body are light grayish brown; white marble patterns are present on ventral surfaces of head, body, and ventral forelimbs, and ventrolateral surfaces of thigh. The white marble patterns are finer and much smaller on ventral head comparing to ventral body, giving a speckle impression; white marble patterns on ventral body are mostly interconnected. A few smaller white spots are also present on ventral thigh, tibia, and femur.

**Coloration of holotype in preservative:** Ornamentation patterns remain after preservation. However, coloration changes after preservation, include: (1) dorsal surfaces of head, limbs, and body become grayish brown, with dark gray patches; (2) lower parts of canthus rostralis and supratympanic fold, transverse bands on dorsal limbs, as well as lower part of lateral body become dark gray; (3) ventral surfaces of throat and limbs become brown, with grayish white spots.

**Variation:** Morphometric variations of the type series are shown in Table 4. Most external morphological characters are identical between the two individuals, but the paratype is smaller than the holotype (SVL 14.6 mm in paratype male vs. 20.4 mm in holotye female), as well as in having rather paler dorsal coloration and more dense spots on ventral body. No secondary sexual characters, such as vocal sac or nuptial pad, are observed in the paratype male, but a single black testicle was observed on the left side of the male paratype, which is oval shaped and relatively large, with a longitudinal length of about 1.5 mm.

**Table 4:** Measurements (in mm) of type series of *Liurana vallecula* sp. nov.

Number	Status	Sex	SVL	HL	HW	SL	IND	IOD	UEW
KIZ014083	Holotype	Female	20.4	7.3	7.9	3.2	3.0	2.2	2.0
Ratio to			–	37.2	38.7	15.6	14.5	10.7	9.8
SVL (%)																
KIZ014106	Paratype	Male	14.6	5.6	6.3	2.5	2.3	1.5	1.5
Ratio to SVL (%)			–	38.1	43.2	17.5	15.7	10.4	10.0
Number	ED	TD	LAHL	LAD	HAL	FML	TL	TFL	FL
KIZ014083	2.9	1.3	9.0	1.5	5.1	10.9	11.8	17.4	11.6
Ratio to SVL (%)	14.1	6.4	44.1	7.3	24.7	53.3	57.6	85.3	57.0
KIZ014106	2.0	1.0	6.3	1.1	3.3	7.7	8.5	11.2	7.1
Ratio to SVL (%)	13.7	43.6	7.4	22.4	52.7	58.4	76.8	49.0	43.6

See methods section for abbreviations. –: Not applicable.

**Ecological and natural history notes:** *Liurana vallecula* **sp. nov.** is a terrestrial, leaf-litter specialist, inhabiting the forest floor of tropical broad-leaf forest at low elevations (below 1 000 m a.s.l.) near Yarlung Zangbo River and its immediate tributaries. The female holotype had about five immature eggs in the left ovary, which were well developed and relatively large.

**Distribution:** Currently the new species is known only from the type localities of Xirang and Maniweng of Beibeng, Medog County, Nyingchi Prefecture, Tibet, China. The new species likely inhabits other nearby regions in southern Tibet (see Discussion below).

**Etymology:** The specific epithet of the new species, “*vallecula*” means “valley inhabitor”, in reference to the habitat of this species in the lower river valley of Yarlung Zangbo Grand Canyon. We suggest Valley Papilla-tongued Frog as its English common name and He Gu She Tu Wa (河谷舌突蛙) as its Chinese common name.

**Comparisons:** *Liurana vallecula* **sp. nov.** differs from the three congeners by having a much wider head (HW/HL >100% vs. <100%) and longer hindlimbs (tibiotarsal articulation reaching beyond tip of snout when adpressed vs. reaching only anterior corner of eye when adpressed). In addition, *Liurana vallecula* **sp. nov.** can be further distinguished from *L. alpina* and *L. xizangensis* by having a smaller body size (SVL 14.6 mm in male, 20.4 mm in female vs. SVL 23.2–24.9 mm in males, 25.5 mm in female for *L. alpina*; 20.6–24.5 mm in males, 29.4–30.5 mm in females for *L. xizangensis*); and from *L. medogensis* by its different ventral pattern (small, marbled patterns or speckles vs. broad, vermiculated stripes).

## DISCUSSION

### *Liurana medogensis* from Southern Tibet

Recently, Borah et al. (2013) reported *L. medogensis* from the eastern part of Southern Tibet, which they recognized as *Limnonectes* (*Taylorana*) *medogensis* in the study (localities 1 and 2 in light blue; Figure 1) (all abbreviations of “*L*.” in this paragraph refer to *Liurana*, not *Limnonectes*). Although one of the reported specimens (ZSI a11549) resembles the external appearance of *L. medogensis* based on the figure in the manuscript (Borah et al., 2013; Figure 1), according to the description, it possesses morphological characteristics that differ from the diagnosis of the genus *Liurana*, including having a distinct vomerine ridge, prominent vomerine teeth, and rudimentary webbing on toes (vs. vomerine ridge, vomerine teeth, and toe webs absent in *Liurana*) (Fei et al., 2009, 2010; Yan et al., 2016). Furthermore, this specimen was collected at a much higher elevation (2 000–2 500 m a.s.l.) than the known range for *L. medogensis* (about 1 400 m a.s.l.). For the second reported specimen of *L.* cf. *medogensis* (BMHE a0081), based on the description provided by the authors, it matches the morphological diagnosis of our new species, *Liurana vallecula*, including having a wide head (HW/HL>100%), but lacks the broad, vermiculate patterns on the ventral surface of the body (Borah et al., 2013). As we cannot examine these specimens or obtain their genetic data, we cannot confirm the taxonomic status of these two specimens reported by Borah et al. (2013). Future taxonomic studies are needed to gain a better understanding of *Liurana* diversity in this region.

### Ecotypes

*Liurana* diversity can be divided into two major ecotypes: the alpine ecotype that inhabits cool, moist mixed forests at 2 000–3 000 m a.s.l. (including *L. alpina* and *L. xizangensis*), and the tropical ecotype that inhabits low-elevation tropical rainforest below 2 000 m a.s.l. (including *L. medogensis* and *Liurana vallecula*). For the first ecotype, coloration of individuals is highly variable, ranging from uniform bright reddish orange to marbled purplish gray. Frogs of this ecotype live under thick layers of moss on fallen tree trunks or rocks along the forest edge. The second ecotype consists of leaf litter specialists that inhabit the forest floor under pristine tropical rainforest. Coloration of this ecotype is much more cryptic, ranging from a single, wide, brownish orange dorsal stripe to dark brown with darker marbling (Figure 4, Figure 5).

Even though *L. medogensis* and *Liurana vallecula* both belong to the second ecotype, they do not form a monophyletic group according to our genetic data (Figure 2). Future ecological studies are needed to further differentiate the ecological niche of each species and investigate the evolution of ecotypes in the genus *Liurana*.

### Reproductive biology

**Figure 6:**
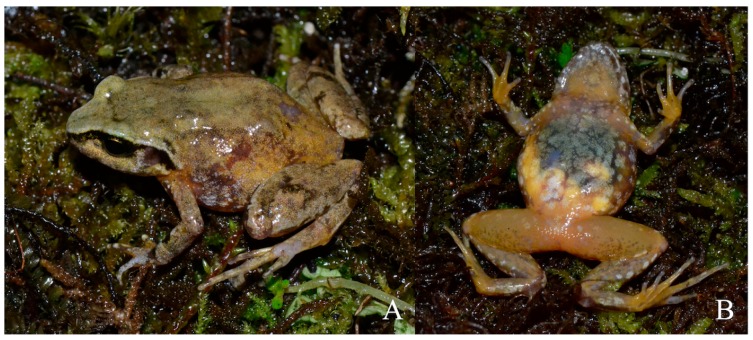
Gravid female (A) and developing eggs through transparent abdomen (B) of Liurana xizangensis from 62K, Medog County, Tibet, China. Photos by Ke Jiang

Although the reproductive biology, particularly the direct developing reproductive mode, has long been documented within the family Ceratobatrachidae (Brown & Alcala, 1982; Siler et al., 2010), little is known about the reproductive biology of *Liurana* specifically. Considering the Tibetan *Liurana* species as *Platymantis* at that time, Hu et al. (1987) first commented on the reproductive mode of the Tibetan species and suggested that they may go through direct development, similar to other *Platymantis* species. It was only until 2010 when the eggs of *Liurana* species in the alpine ecotype from Tibet were first collected (Li et al., 2010). According to Li et al. (2010), eggs of the unidentified *Liurana* species from 62K in the Medog County (confirmed as *L. xizangensis* according to Yan et al. (2016) and the present study) were large (with a diameter of about 3.5 mm) and the clutch size was small. These egg characteristics are similar to those of direct development in the same family (Siler et al., 2010). Similar to Li et al. (2010), we also observed eggs in a female *L. xizangensis* (KIZ014153) from the same locality, where the female displayed 14 and 16 yellowish white eggs in the left and right oviduct, respectively, though not all were fully developed (Figure 6).

More recently, Borah et al. (2013) provided information regarding the reproductive biology of the tropical ecotype of *Liurana* from Southern Tibet. Embryos displayed characteristics of the direct developing species in the family Ceratobatrachidae, including having a few embryos, large individual embryo size, and whitish coloration (Borah et al., 2013). Based on these similarities, the authors claim that this *Liurana* species reproduces through direct development without the larval stage (Borah et al., 2013). However, such conclusions on direct development in *Liurana* are still based on indirect inferences of egg characteristics. Therefore, we recommend future studies focus on the reproductive biology of the genus to clarify the reproductive mode of *Liurana*.

### Acoustic signals

In addition to the differences in general habitat preferences and external morphology, the two ecotypes of *Liurana* also differ in their acoustic signals. In fact, extensive acoustic signals have been observed in the alpine ecotype only (Fei et al., 2009; Hu et al., 1987; present study). Such differences in acoustic signals may be explained by the relative cost-benefit ratios of calling in each specific environment (Ryan, 1988). Future ecological and behavioral studies are needed to confirm this hypothesis in the field and investigate the communication strategies of the tropical ecotype.

### Evolution

Frogs of the genus *Liurana* are endemic to eastern Himalaya, and all are found in the Yarlung Zangbo Drainage in Southern Tibet (Figure 1). Based on the available distribution data, *L. xizangensis* is distributed on the eastern side of the Yarlung Zangbo River, whereas *L. alpina* is found on the western side of the river. As the two species are found in cool environments at high elevation only (>2000 m a.s.l.), the hot tropical valley of the Yarlung Zangbo River may serve as a dispersal barrier, thereby shaping the genetic diversity and facilitating the speciation processes of the two congeners. This hypothesis is partly supported by our genetic data, where closely distributed populations from two sides of the river possessed higher genetic divergence than further distributed populations from the same side (Table 3). Future studies should expand population sampling of the two species along both sides of Yarlung Zangbo River to examine its role in the evolution of *Liurana*.

### Conservation

As micro-endemic habitat specialists, *Liurana* species are threatened by habitat destruction in southern Tibet. Based on our continuous field surveys since 2012, considerable habitat destruction has been observed at 62K in Medog County, which is one of only three known localities for *L. xizangensis*. Unregulated infrastructure developments have destroyed the mossy fields along the forest edges, streams, and wetlands, which constitute the core habitats not only for *L. xizangensis*, but also other micro-endemic anuran species such as *Scutiger wuguanfui* and *S. spinosus* (Jiang et al., 2016). Similarly, continuous tourist development and road construction along the hiking trail of Medog pose serious threats to habitat at the only known locality of *L. alpina*. Therefore, we recommend that local authorities and regional governments take habitat conservation into account when making developmental decisions, and we urge law enforcement agencies to enforce the existing environmental regulations of construction projects in the region, particularly in Medog County.

## NOMENCLATURAL ACTS REGISTRATION

The electronic version of this article in portable document format will represent a published work according to the International Commission on Zoological Nomenclature (ICZN), and hence the new names contained in the electronic version are effectively published under that Code from the electronic edition alone (see Articles 8.5–8.6 of the Code). This published work and the nomenclatural acts it contains have been registered in ZooBank, the online registration system for the ICZN. The ZooBank LSIDs (Life Science Identifiers) can be resolved and the associated information can be viewed through any standard web browser by appending the LSID to the prefix http://zoobank.org/.

Publication LSID:

urn:lsid:zoobank.org:pub: 8D6905B9-0AEA-4091-BCA6-A7C849BD1848.

Liurana vallecula LSID:

urn:lsid:zoobank.org:act: 2AD61C3A-E263-46CC-A919-399992A558C8.

## APPENDIX I

Specimens examined for comparison. Museum abbreviations follow those from the methods section.

*Liurana alpina* (*n*=4): KIZ011140–42 (males), KIZ07358 (Dayandong (type locality)) Medog County, Tibet, China.

*Liurana medogensis* (*n*=3): KIZ010955, 05886, 05587 (males), (Xigong Lake (type locality)) Medog County, Tibet, China.

*Liurana xizangensis* (*n*=11): KIZ012704, 014046 (males), (Tongmai (close to type locality “Yi’ong”, =Yigong)) Bomê, Tibet, China; KIZ06707, 09954–56, 011105–06, 012706 (males); KIZ09953, 014153 (females), 62K, Medog County, Tibet, China.

## APPENDIX II

**Table:** GenBank sequences used and accession Nos. of novel sequences

				Gene and GenBank accession No.			
Genus	Species	Voucher or tissue number	Locality	*COI*	*Rag1*	*Rod*	*Tyr*
*Alcalus*	*tasanae*	CAS232349	Myanmar	–	KU243096	KU243106	KU243116
*Alcalus*	*tasanae*	CAS247243	Myanmar	–	KU243097	KU243107	KU243117
*Alcalus*	*tasanae*	THNHM20534	Myanmar	–	KU243098	KU243108	KU243118
*Ceratobatrachus*	*guentheri*	VUB1017(SR5543)	Solomon Islands	AY883979	DQ347272	DQ347391	DQ347179
*Cornufer*	*boulengeri*	BPBM22329	Papua New Guinea	HQ844999	KP298309	–	KP298385
*Ingerana*	*tenasserimensis*	CAS205064/ TADP918	Myanmar/Thailand	KR087736	DQ347258	AY322236	KP298327
*Limnonectes*	*limgorgi*	VUB1218	Laos	–	DQ347286	DQ347407	DQ347194
*Liurana*	*alpina*	KIZ011140	Medog, Tibet, China	MK462138	KU243094	KU243104	KU243114
*Liurana*	*alpina*	KIZ011141	Medog, Tibet, China	MK462139	KU243095	KU243105	KU243115
*Liurana*	*alpina*	KIZ07357	Medog, Tibet, China	MK462137	–	–	–
*Liurana*	*medogensis*	KIZ010955	Medog, Tibet, China	MK462136	–	KU243103	KU243113
*Liurana*	*vallecula*	KIZ014083	Medog, Tibet, China	MK462134	MK315115	MK462150	MK462155
*Liurana*	*vallecula*	KIZ014106	Medog, Tibet, China	MK462135	–	MK462151	MK462156
*Liurana*	*xizangensis*	KIZ06707	Medog, Tibet, China	MK462140	KU243092	KU243101	KU243111
*Liurana*	*xizangensis*	KIZ09956	Medog, Tibet, China	MK462141	–	–	–
*Liurana*	*xizangensis*	KIZ011104	Medog, Tibet, China	MK462144	–	–	–
*Liurana*	*xizangensis*	KIZ012705	Medog, Tibet, China	MK462142	–	–	–
*Liurana*	*xizangensis*	KIZ012706	Medog, Tibet, China	MK462143	–	–	–
*Liurana*	*xizangensis*	KIZ012707	Medog, Tibet, China	MK462145	–	–	–
*Liurana*	*xizangensis*	KIZ012704	Medog, Tibet, China	MK462146	–	–	–
*Liurana*	*xizangensis*	KIZ014046	Medog, Tibet, China	MK462147	–	–	–
*Platymantis*	*hazelae*	TNHC62160 & CMNH-RSK3918	Phillipines	–	DQ347248	DQ347369	DQ347153

–: Not available.
